# Attention-enhanced SAM with PBFO tuning: advancing glioma MRI segmentation

**DOI:** 10.3389/fmed.2026.1730353

**Published:** 2026-02-19

**Authors:** Salem Alhatamleh, Hamad Yahia Abu Mhanna, Mohammad Amin, Amal Alishwait, Mohammad Latayfeh, Qutaiba Mohammad, Ghada A. Khouqeer, Abdullah Alrefai, Sitah Alanazi, Kholoud J. Sandougah

**Affiliations:** 1 Department of Computer Science, Faculty of Information Technology and Computer Sciences, Yarmouk University, Irbid, Jordan; 2 Department of Medical Imaging, Faculty of Allied Medical Sciences, Isra University, Amman, Jordan; 3 Faculty of Medicine, Yarmouk University, Irbid, Jordan; 4 Department of Physics, College of Science, Imam Mohammad Ibn Saud Islamic University (IMSIU), Riyadh, Saudi Arabia; 5 Faculty of Medicine, Jordan University of Science and Technology, Irbid, Jordan; 6 Department of Radiology, College of Medicine, Imam Mohammad Ibn Saud Islamic University (IMSIU), Riyadh, Saudi Arabia

**Keywords:** deep learning, glioma segmentation, lower grade gliomas, medical image analysis, MRI imaging, segment anything model (SAM)

## Abstract

**Introduction:**

The segmentation of brain tumor MRI images is one of the most challenging tasks because of the variability and complexity associated with tumor tissues. This study introduces PoSAM-ULTRA, an improved segmentation framework designed to enhance the accuracy and robustness of brain tumor segmentation.

**Methods:**

PoSAM-ULTRA employs the Polar-Bear Foraging Optimisation (PBFO) algorithm for hyperparameter tuning and utilizes an improved Segment Anything Model as its backbone. The framework is based on a ResNet-34 encoder modified to accept a four-channel input (RGB + prior information). Multi-scale feature extraction is performed via DownBlocks, while discriminative feature learning is enhanced using the Convolutional Block Attention Module (CBAM). Attention Gates are incorporated to ensure effective skip connections, and a multistage decoder is used for robust upsampling and feature integration. The model was evaluated on a dataset from the Integrative Genomic Analysis of Diffuse Lower Grade Gliomas (LGG) and compared with UNet, UNet++, and nnUNet.

**Results:**

The proposed PoSAM-ULTRA model outperformed the baseline models, achieving superior performance with a Dice score of 91.4%, IoU of 88.9%, Accuracy of 99.8%, Precision of 95.2%, and Recall of 93.3%.

**Discussion:**

The obtained results demonstrate the robustness and reliability of PoSAM-ULTRA in handling the complexity of brain tumor MRI segmentation, highlighting its effectiveness for challenging medical image segmentation tasks.

## 1 Introduction

Brain tumors are not the most prevalent cancers in the world, but they are among the ones with the highest burden based on their adverse impact on neurological function, cognitive deterioration, and overall morbidity and mortality. Their neoplasias are heterogeneous with tumors of varying malignant potential, biological behavior, and clinical course. According to the latest figures provided by the central brain tumor registry of the United States (CBTRUS), over 1.3 million people nationwide have been diagnosed with a primary brain or Central nervous system (CNS) tumor, and up to 85% are non-malignant. However, malignant tumors of the brain, particularly Glioblastoma Multiforme (GBM), have some of the poorest survival rates among all types of cancer (1). Age plays an essential role in the histological, biological, and prognostic profile of the brain tumor: Glioblastoma has a median age of occurrence at 64 years, with the highest incidence in people aged 75–84 (2). The term “low-grade glioma (LGGs)” generally refers to diffusive gliomas of the brain that are more aggressive than Grade I but less aggressive than Grade IV (glioblastomas). These include WHO Grade II (“low grade”) and Grade III (“anaplastic” or high-intermediate grade) gliomas (3). Traditionally, gliomas have been classified based on histopathology (cell shape, mitoses, etc.) (4). And more recently (since about 2016 and as applied by WHO CNS5, 2021), molecular markers are required components of classification (5).

Imaging is often the very first step raising suspicion of a brain tumor, especially when patients come in with neurological symptoms such as seizures, headache, or changes in cognitive functions. In the case of WHO Grade II and III diffuse gliomas, which are also referred to as LGGs, magnetic resonance imaging (MRI) is the primary method not only for diagnosis but also for monitoring over a long period (3). Along with the current advances in imaging and molecular diagnostics, the separation of lower-grade gliomas (LGG) from high-grade gliomas (HGG) or other brain lesions continues to be a major clinical hurdle. Accurate segmentation (delineation of tumors on medical images) becomes critical for the diagnosis, progression monitoring, and treatment planning of LGGs. Underestimating the tumor size might lead to incomplete resection and quicker recurrence, while overestimation could mean that healthy brain tissue, especially around the eloquent areas, is destroyed (5). Manual segmentation is not only a very lengthy process but also highly subjective, particularly when different observers are involved (5). The differences in tumor delineation may pose a risk to the reproducibility of study results.

CNNs in capturing the spatial characteristics and context information which is why they are very suitable for the detection of non-cancerous tumors in delicate and challenging areas such as the brain. Although AI is a great help, it not only saves the time that is usually taken to perform the task manually but also the orderly and consistent results which are the essentials in crucial health decisions and long-term monitoring. The availability of public datasets like BraTS (Brain Tumor Seg) has greatly accelerated the adoption and validation of CNN-based models. Thus, the AI industry is the one that takes the major and fast-growing part in medical imaging studies. Over the last 10 years, the focus of research shifted toward the segmentation of small brain tumors in simple gliomas. This was achieved mainly due to the drastic increase in the availability of annotated data sets and the improvement of skills. Numerous studies have proven that AI tools, predominantly employing CNNs & U-Net architectures, can be extremely accurate. They are often on par or superior to human performance in specific cases. These tools not only excel in marking tumor boundaries but also in differentiating small parts such as the tumor core, edema, and hot areas. Additionally, recent studies have applied a great number of MRI modalities (T1, T1c, T2, FLAIR) & high-resolution techniques. Moreover, 3D modeling has been used for these processes. This has contributed to the understanding of the blend and variety in LGGs. Many such initiatives have already been tested in older medical databases, and more are now being tested in new ones. This suggests that they are indeed effective in real-world applications. They assist in treatment and monitoring.

Brain tumor segmentation in magnetic resonance imaging (MRI) is a fundamental step in supporting accurate diagnosis and effective treatment planning. However, the high variability in tumor shape and size, coupled with the noise in medical images, makes segmentation a significant challenge for traditional models. Hence, the a need to develop more accurate and flexible models capable of handling these complexities. This study aims to develop a new model capable of improving the accuracy and reliability of brain tumor segmentation by combining advanced optimization strategies with modern deep learning architectures. The core concepts of PoSAM-ULTRA in glioma MRI image segmentation aim to achieve functional integration and adaptation of existing components, centered around three key innovations: First, the SAM model backbone has been re-engineered to accept four-channel inputs for co-processing RGB and tumor-related information, enriching feature context and improving tumor boundary definition. Second, an enhanced, attention-coordinated feature fusion mechanism has been introduced, incorporating CBAM modules and attention gates into multiscale encoding and interpretation blocks to enhance the differentiation of low-contrast and irregular glioma regions while maintaining structural consistency. Third, the PBFO hyperparameter fusion process represents a novel optimization strategy within medical SAM segmentation pathways, providing more stable convergence and improved autogeneralization. This combination of three elements results in a coherent and specialized enhancement for glioma segmentation. This will contribute to enhancing overall performance compared to popular models such as UNet, UNet++, and nnUNet.

The main contributions of this research can be summarized as follows:

- Proposing the PoSAM-ULTRA model, which combines the Polar-Bear Foraging Optimization (PBFO) algorithm to fine-tune hyperparameters with the Segment Anything Model (SAM) as the underlying segmentation architecture.
- Designing an improved multi-level feature extraction, incorporating attention mechanisms such as CBAM and Attention Gates to optimize the use of lateral connections and eliminate irrelevant information.
- Adopting a deep supervision mechanism (Deep Supervision) with a complex loss algorithm (BCE, Dice, Focal) to enhance the learning process and achieve a better balance between sensitivity and accuracy.
- Evaluating the model on the LGG dataset demonstrated its superiority over well-known models in brain image segmentation, achieving the highest performance in Dice, IoU, precision, and recall.

Section 2 discusses the techniques used, providing a comprehensive explanation of the dataset, the structure of the proposed approach, and the training methods. Section 3 analyzes the data and evaluates the effectiveness of the proposed PoSAM-ULTRA model in brain tumor segmentation tests. Section 4 reviews key studies in brain tumor segmentation and compares them to the proposed approach. Section 5 concludes with conclusions and recommendations for further research.

## 2 Methodology

**FIGURE 1:**
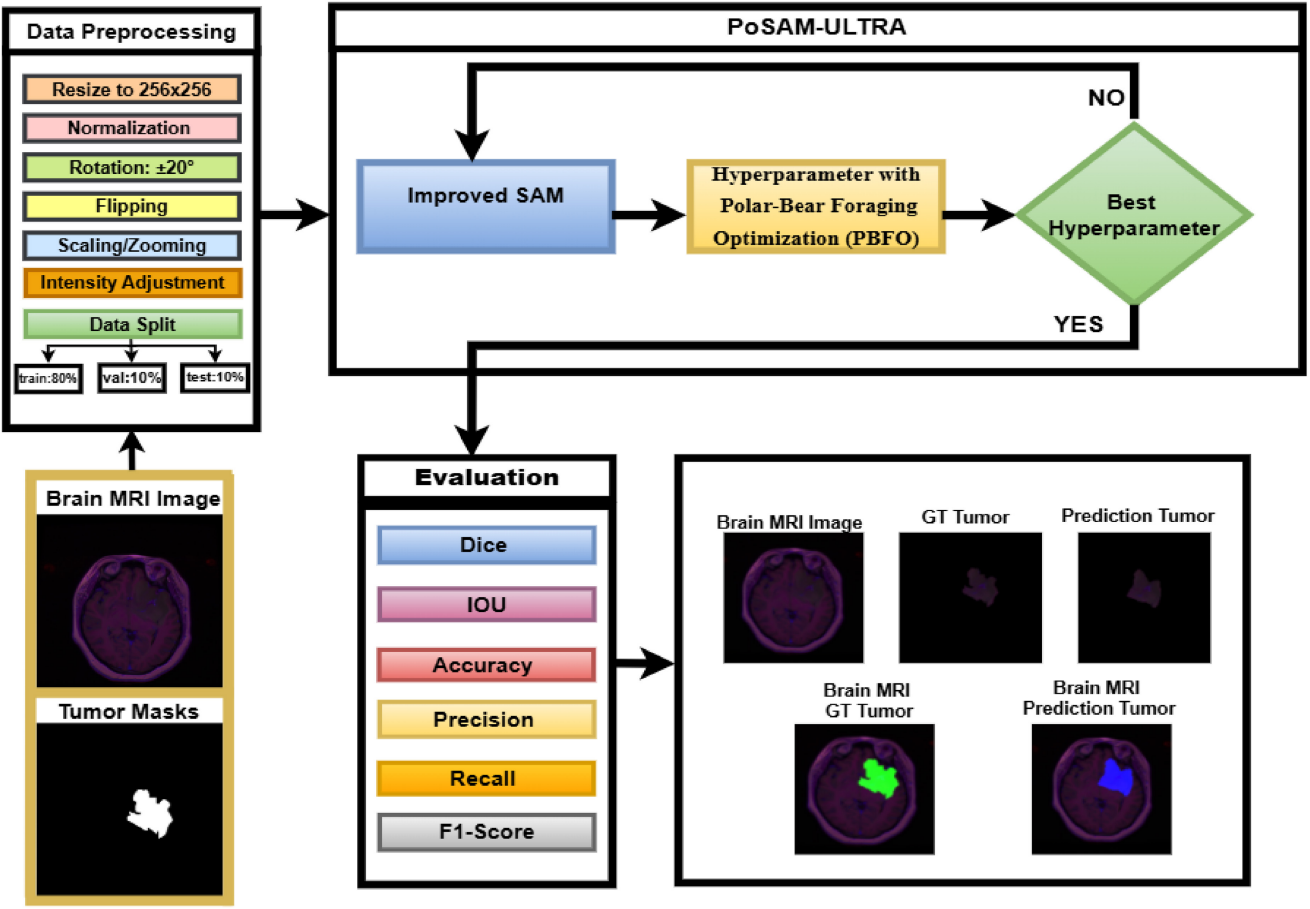
Overview of the proposed PoSAM-ULTRA framework for glioma segmentation.

In this study, we propose PoSAM-ULTRA, a novel deep learning framework for brain tumor segmentation. The method integrates the Segment Anything Model (SAM) as a backbone with a ResNet34-based 4-channel encoder, enhanced by CBAM attention modules, extended skip connections, and deep supervision through auxiliary outputs, as shown in Figure 1. On the performance side, PBFO hyperparameter search is performed, greatly minimizing tuning expenses. The dataset used is the Integrative Genomic Analysis of Diffuse Lower-Grade Gliomas (LGG) collection composed of paired MRI scans and tumor masks. Image preprocessing is done by resizing, normalizing, and augmenting for better robustness. Then the final models are trained for 50 epochs using the configurations derived from the optimization technique. Performance measurement itself takes place with an extended list of evaluation criteria, namely, Dice, IoU, Precision, Recall, Accuracy. Baselines of U-Net, U-Net++, and nnU-Net-lite serve as comparison points for PoSAM-ULTR.

### 2.1 Dataset

**FIGURE 2:**
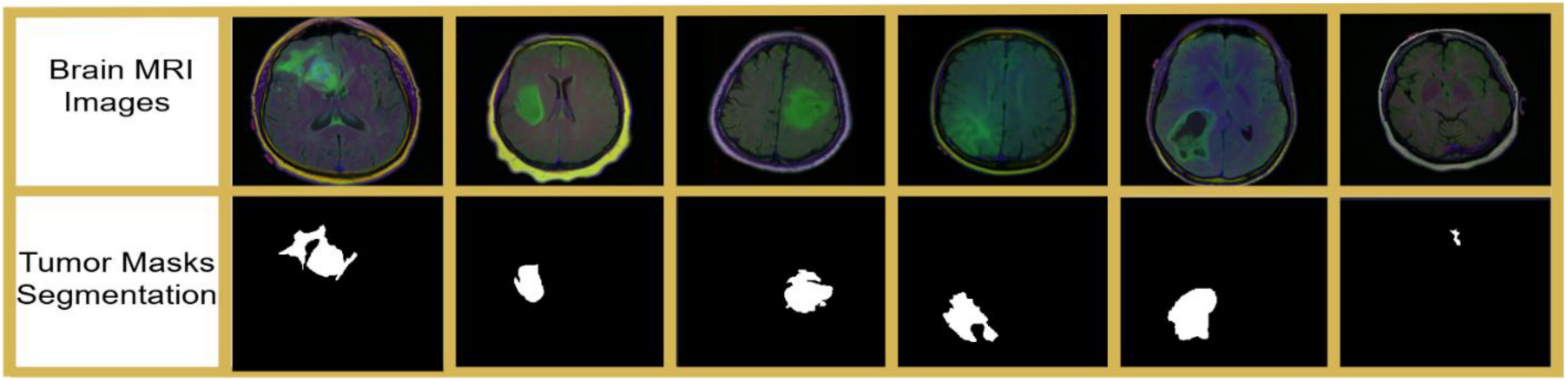
Brain MRI images with ground truth tumor masks.

The LGG Segmentation Dataset is a publicly accessible database of MRI brain scans for lower-grade gliomas, originally released by Mazurowski et al. (6). It involves MR images with manually provisioned FLAIR abnormality segmentation masks that allow for the exact delineation of chromatic abnormality regions. The dataset includes data from 110 patients from The Cancer Genome Atlas (TCGA) LGG collection, all of whom have FLAIR sequences and associated genomic cluster information. In addition to the images, patient metadata and genomic tumor cluster information are included in a separate CSV file for integrated studies of imaging and genomic characteristics. The MRI scans are from The Cancer Imaging Archive (TCIA), providing a set of standardized and publicly available imaging data. This data has been used to investigate the associations between tumor shape characteristics, as determined by deep learning, and a tumor’s genomic subtype, as well as the effect size of these associations on patient outcomes. Figure 2 shows an example from the LGG Segmentation Dataset.

### 2.2 Data preprocessing

Preprocessing medical images is of utmost importance to maintain data in a standardized form, ensuring all properties of robustness and suitability for deep-learning models. In PoSAM-ULTRA, the entire pipeline of preprocessing steps was designed, encompassing resizing, normalization, data augmentation, and dataset partitioning, of MRI scans and their respective tumor masks of the LGG dataset (7). These steps ensure standard input dimensions, minimize intensity variability between subjects, increase training examples, and allow for unbiased evaluation. Resizing since an MRI scan naturally differs in resolution and aspect ratio, both images and their respective masks were resized to a uniform resolution of 256 × 256 pixels guaranteeing compatibility of all inputs with the encoder-decoder-style PoSAM-ULTRA, while simultaneously providing adequate spatial detailing for precise detections of tumor boundaries. *I* represent the original MRI image (8). One gets the corresponding ground mask resized with the same transformation to maintain spatial alignment, as shown in Equation 1.


$$I_{r\,e\,s\,i\,z\,e\,d}=R\,e\,s\,i\,z\,e\,\left(I,256\times 256\right)$$ (1)

Normalized medical images often suffer from varying intensity distributions due to differences in acquisition protocols and patient-specific contrast variations (9). To stabilize the training process and accelerate convergence, pixel values were normalized to the range [0, 1] using min-max scaling *I*_*min*_ and *I*_*max*_ denote the minimum and maximum pixel intensities of the image, respectively. This normalization ensures that the input dynamic range is consistent across all subjects, allowing the network to focus on structural differences rather than intensity biases, as shown in Equation 2.


$$I_{n\,o\,r\,m}=\frac{I-I_{m\,i\,n}}{I_{m\,a\,x}-I_{m\,i\,n}}$$ (2)

Data augmentation to improve generalization and reduce overfitting, extensive on-the-fly augmentation was applied during training (10). Augmentation increases the diversity of the dataset by simulating variations in patient anatomy and acquisition conditions. The following transformations were randomly applied. Rotation random angular rotations up to ± 20, simulating changes in patient orientation. Flipping: Random horizontal and vertical flips; please enforce symmetrical invariance (11). Scaling/Zooming small random rescaling of images, making the training robust toward changing tumor sizes. Intensity Adjustment changes brightness and contrast to simulate changes in imaging conditions. Formally, the augmented dataset is defined as *D* is the original dataset, *M* is the mask, and 𝒯 is the distribution of transformations applied. This guarantees that the model sees more diverse tumor appearances, which is critical for robust segmentation of low-grade gliomas, as shown in Equation 3.


$$D_{a\,v\,g}=\left\{T\,\left(I,M\right)|\left(I,M\right)\in D,T∼T\right\}$$ (3)

The dataset was divided into 80% training (3,143 images), 10% validation (393 images), and 10% testing (393 images) to ensure a fair and unbiased assessment of performance. The training set *D*_*train*_ is used to optimize model weights via backpropagation. The validation set *D*_*val*_ is used for hyperparameter tuning and observing model convergence. The test set *D*_*test*_ is reserved for the final evaluation to get a measure of conscious ability as shown in Equation 4.


$$D=D_{t\,r\,a\,i\,n}\cup D_{v\,a\,l}\cup D_{t\,e\,s\,t},\left|D_{t\,r\,a\,i\,n}\right|=0.8\,\left|D_{v\,a\,l}\right|=0.1\,\left|D_{t\,e\,s\,t}\right|=0.1$$ (4)

To generate the four-channel inputs for the SAM backbone, a prior-information map is incorporated to highlight glioma probability regions. This prior-map is generated via a lightweight preprocessing pipeline that begins with skull-stripping using HD-BET and image intensity normalization using a Z-score. A primary tumor probability map is then produced through unsupervised clustering (K-means with $k = 3$), where the cluster with the highest average intensity is considered the primary tumor candidate. This mask is refined using morphological closures and a median filter to reduce noise, and then min–max normalization to fit the range [0, 1]. This prior-channel approach has proven to provide meaningful spatial semantics, confirming that it provides additional structural cues that identify potential tumor regions without replacing the detailed learning process of the model.

A brain tumor dataset is, generally, characterized by extreme class imbalance, for tumor-region image pixels make up a much smaller proportion of the total image than do background pixels. To confront this, the weights of the loss function were optimized through PBFO to maintain an equilibrium between Dice loss and binary cross-entropy (BCE). Thus, the model is made to be sensitive to the tumor of small structures, while at the same time avoiding any chance of its overbearing influence by background pixels. The preprocessing pipeline allowed for the standardization, diversification, and balancing of the dataset from which PoSAM-ULTRA can draw worthy inputs. These would have helped directly in accurate tumor boundary detection, patient-to-patient generalization, and reduction of overfitting of the model so that it can perform well in segmentation against the baseline methods.

### 2.3 Improved segment anything model

**FIGURE 3:**
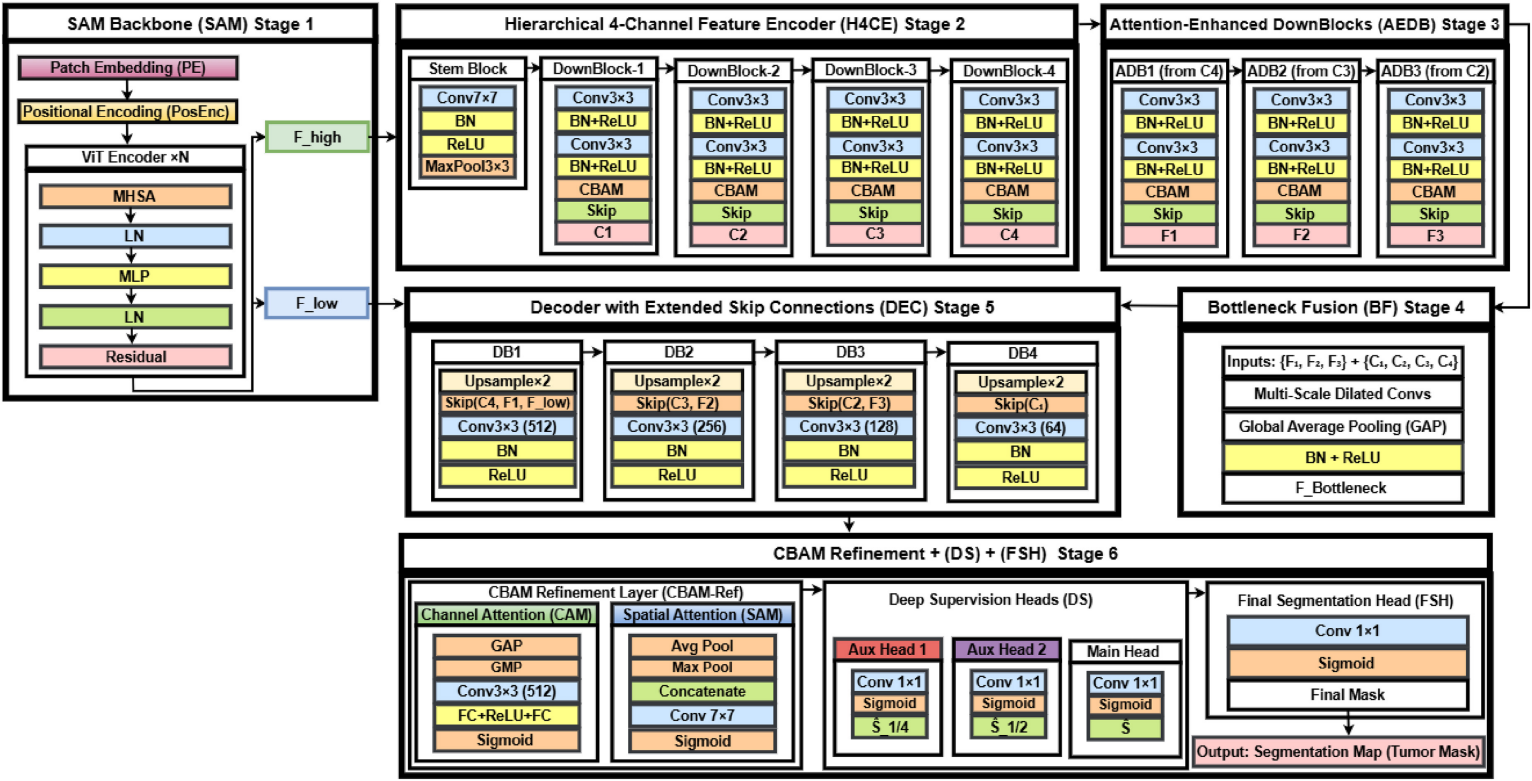
Architecture overview of the six-stage improved SAM-based segmentation framework (PoSAM-ULTRA).

The method integrates Segment Anything Model (SAM) as a backbone with a ResNet34-based 4-channel encoder, enhanced by CBAM attention modules, extended skip connections, and deep supervision through auxiliary outputs. To optimize performance, we employ the Polar-Bear Foraging Optimization (PBFO) algorithm for efficient hyperparameter search (Figure 3).

#### 2.3.1 Segment anything model

The Segment Anything Model (SAM) serves as the primary backbone of PoSAM-ULTRA, providing powerful feature extraction for MRI brain tumor images (12). SAM is designed to capture both global and local structures, allowing the model to identify fine-grained tumor regions while preserving spatial context. Given an input image *I* ∈ ℝ^C H W^, SAM computes hierarchical feature maps *F*^(*l*)^ at multiple layers *l* using convolutional operations, as shown in Equation 5.


$$F^{\left(l\right)}=\sigma \,\left(W^{\left(l\right)}*F^{\left(l-1\right)}+b^{\left(l\right)}\right),F^{\left(0\right)}=I$$ (5)

Where * denotes convolution, *W*^(*l*)^ and *b*^(*l*)^ are the weights and biases of layer *l*, whereas σ is a nonlinear activation function like ReLU and GELU (13). In previous layers, the feature maps assimilate gradual low-texture details and in deeper layers, they integrate high-level semantic information. SAM incorporates an attention mechanism to improve feature relevance by emphasizing salient regions in the data. Let M_*c*_(F) and M_*s*_(F) be the channel and spatial attention maps, and the attended features are, as shown in Equation 6.


$$F^{\prime }=F\times M_{c}\,\left(F\right)\times M_{s}\,\left(F\right)$$ (6)

This selective weighting enables the backbone to focus on tumor areas and discourage irrelevant areas from the background. Down sampling across layers is applied to reduce spatial resolution and to allow for receptor field growth by way of max pooling or stride convolutions (14). Finally, SAM further generates multi-level feature maps {*F*^(*l*_1_)^,*F*^(*l*_2_)^,*F*^(*l*_3_)^} that are fed into the Res34LikeEncoder4ch and downstream decoder, thereby providing a rich representation for further segmentation processing, as shown in Equation 7.


$$F^{\left(l+1\right)}\,D\,o\,w\,n\,\left(F^{\prime ,\left(l\right)}\right)\,C\,o\,n\,v\,S\,t\,r\,i\,d\,e\,2\,\left(F^{\prime ,\left(l\right)}\right)$$ (7)

#### 2.3.2 Hierarchical 4-channel feature encoder

The encoder from PoSAM-ULTRA is a four-channel convolutional neural network inspired by ResNet34 that extracts hierarchical features from multimodal MRI data (15). The encoder input, *X* ∈ ℝ^4H W^), has three MRI modalities and one prior probability map as channels, each giving complementary structural and tumor-specific information. The stem layer applies a 7 × 7 convolution plus batch normalization and ReLU activation, as shown in Equation 8.


$$F_{0}=R\,e\,L\,U\,\left(B\,N\,\left(C\,o\,n\,v_{7\times 7}\,\left(x\right)\right)\right)$$ (8)

A max-pooling operation halves each spatial dimension for a first-level feature map *F_1_*. Then the encoder receives *F_1_* into four hierarchical DownBlocks, wherein each DownBlock entails two consecutive 3 × 3 convolutions with batch normalization and ReLU activation, and optionally a stride for downsampling to reduce spatial resolution (16). The four levels of feature maps *C*_1_,*C*_2_,*C*_3_,*C*_4_ are produced and denote increasingly abstract and semantically rich representations of the input. These hierarchical features will be used later in the skipping connections to the decoder for localizing tumor regions with high accuracy. The encoder can thus be formulated mathematically as follows, as shown in Equations 9, 10.


$$F_{l+1}=R\,e\,L\,U\,\left(B\,N\,\left(C\,o\,n\,v_{3\times 3}\,\left(F_{l}\right)\right)\right),l=1,\cdots ,4$$ (9)

$$\left(C_{1},C_{2},C_{3},C_{4}\right)=E\,n\,c\,o\,d\,e\,r\,\left(X\right)$$ (10)

Each *C*_*i*_ ∈ ℝ^*F*_*i*_ *H*_*i*_ *W*_*i*_^ corresponds to increasingly abstract and semantically rich representation of the input data. These hierarchical features are then passed to the decoder through skip connections, allowing correct localization of tumor areas. Hence, with this hierarchical design, PoSAM-ULTRA can effectively balance global context with local tumor localization for the challenging lower-grade glioma segmentation task.

#### 2.3.3 Attention-enhanced downblock

The Attention-Enhanced DownBlock forms the fundamental processing unit of the encoder in PoSAM-ULTRA, integrating three components a basic convolutional block conv_block, progressive spatial reduction through the DownBlock, and adaptive feature refinement via the Convolutional Block Attention Module (CBAM) (17). Ultimately, these features allow for effective multi-scale feature learning while also improving the network ability to emphasize the tumor-relevant areas. At its core, each DownBlock begins with a convolutional block that consists of three sequential operations: convolution, followed by batch normalization and then ReLU activation. Given the input feature map *F* ∈ ℝ^C H W^, it performs a basic but effective operation that adapts the local spatial patterns and then prepares the local spatial pattern features for a step of another layer of hierarchy even deeper in the neural network, as shown in Equation 11.


$$F^{\prime }=R\,e\,L\,U\,\left(B\,N\,\left(C\,o\,n\,v_{3\times 3}\,\left(F\right)\right)\right)$$ (11)

The DownBlock stacks two conv blocks consecutively and optionally downsamples by stride to reduce spatial resolution and increase feature channels. The idea is to let the network alternately capture higher semantic information, where *s*1 means feature refinement at the same resolution and *s*2 implies spatial down sampling of 2 times. A hierarchical stacking of DownBlocks across encoder stages maintains a balance between fine-grained localization and contextual abstraction, as shown in Equation 12.


$$F_{l+1}=R\,e\,L\,U\,\left(B\,N\,\left(C\,o\,n\,v_{3\,3}\,\left(F_{l},s\right)\right)\right),s\in 1,2$$ (12)

To increase the encoder representational capacity in PoSAM-ULTRA CBAM is embedded within the convolutional layers (18). CBAM applies channel attention and then spatial attention, which lets the network adaptively refine the features by concentrating on the most discriminating regions of brain tumor MRI scans. Channel attention: Given an intermediate feature map *X* ∈ ℝ^C H W^, CBAM first infers a channel attention map by aggregating spatial information using both global average pooling and global max pooling, and W_0_ & W_1_ are learnable weights, σ is the ReLU activation, and σ is the sigmoid function. The refined feature is obtained, as shown in Equations 13, 14.


$$M_{c}\,\left(F\right)=\sigma \,\left(W_{1}\,\left(\delta \,\left(W_{0}\,\left(A\,v\,g\,P\,o\,o\,l\,\left(F\right)\right)\right)\right)+W_{1}\,\left(\delta \,\left(W_{0}\,\left(M\,a\,x\,P\,o\,o\,l\,\left(F\right)\right)\right)\right)\right)$$ (13)

$$F_{c}=M_{c}\,\left(F\right)\times \text{F}$$ (14)

Spatial attention emphasizes tumor-relevant regions within each feature map. Average pooling and max pooling are applied along the channel axis, and the two outputs are concatenated to form a spatial descriptor, and *f*^7 7^ denotes a convolution with kernel size 7 × 7. The spatially refined feature is then computed (19). The output of CBAM is the sequentially refined feature map. By combining channel-wise and spatial-wise attention, CBAM enables PoSAM-ULTRA to suppress irrelevant background signals and highlight tumor boundaries, which is particularly beneficial for segmenting low-contrast glioma regions, as shown in Equations 15, 16.


$$M_{s}\left(F_{c}\right)=\sigma \left(f^{7\times 7}\left(\left[AvgPool\left(F_{c}\right)\right];;MaxPool\left(F_{c}\right)\right]\right))$$ (15)

$${\hat{F}}_{l+1}=M_{s}\,\left(F^{\prime }\right)\times F_{c}$$ (16)

Combining convolutional feature extraction with progressive downsampling and CBAM-based adaptive attention turns the Attention-Enhanced DownBlock into the most effective discriminant for PoSAM-ULTRA in differentiating tumors from neighboring brain tissues. This design choice places more emphasis on global context relative to local boundary precision, of utmost importance for glioma segmentation. Progressive downsampling reduces computations by shrinking the feature map sizes at deeper layers.

#### 2.3.4 Decoder with extended skip connections

The decoder in PoSAM-ULTRA performs the task of reconstructing the high-resolution segmentation mask from the multi-scale hierarchical features extracted by the encoder (20). Different from common decoders, skip connections extended beyond commonplace lengths are used to retain fine spatial details from the encoder, which is crucial in drawing tumor boundaries accurately in MRI scans. At a particular stage of the decoder, the provided lower-resolution feature map $F_{d}^{\left(l+1\right)}\in {\mathbb{R}}^{\text{C}\,\text{H}\,\text{W}}$ is first upsampled, normally, by bilinear interpolation and transposed convolution (21), to the spatial dimensions of the corresponding encoder feature map *C_l_*, as shown in Equation 17.


$$F_{d}^{\left(l\right)}=U\,p\,s\,a\,m\,p\,l\,e\,\left(F_{d}^{\left(l+1\right)}\right)$$ (17)

The upsampled feature map is then concatenated with the encoder feature maps from all previous stages through extended skip connections. This concatenation enables the decoder to benefit from both the low-level spatial information and the high-level semantic feature information at the same time, which is useful for improving localization of small and irregular tumors, as shown in Equation 18.


$$F_{d}^{\left(l\right)}=C\,o\,n\,v_{3\times 3}\,\left(\left[F_{d}^{\left(l\right)},C_{l},C_{l-1},\cdots ,C_{1}\right]\right)$$ (18)

In the decoder, attention integration of the CBAM by (22) attention applied in the encoder is similarly implemented in a refinement step in the decoder on each concatenated feature block to enable attention to tumor-related regions and suppression of features that are unrelated to the tumor in the background (23). Progressive reconstruction then involves the decoder performing multiple stages of up sampling, concatenation, and attention refinement until the feature map reaches the original input resolution HW. The final segmentation output *S* ∈ ℝ^1H W^ is obtained through a 11convolution followed by a sigmoid activation, as shown in Equations 19, 20.


$${\hat{F}}_{d}^{\left(l\right)}=C\,B\,A\,M\,\left(F_{d}^{\left(l\right)}\right)$$ (19)

$$S=\sigma \,\left(C\,o\,n\,v_{1\times 1}\,\left({\hat{F}}_{d}^{\left(1\right)}\right)\right)$$ (20)

The decoder can reconstruct a segmentation mask that is spatially precise and informed semantically owing to extended skip connections with CBAM refinement at every stage. Consequently, this design enables PoSAM-ULTRA to accurately segment the lower-grade glioma, capturing delicate boundaries of the tumors that standard encoder-decoder architectures lose.

#### 2.3.5 Deep supervision

In PoSAM-ULTRA, deep supervision introduces additional outputs to represent intermediate layers of the decoder to support gradient flow and to stabilize learning (24). With intermediate predictions, the model receives more influences during backpropagation, leading to improved learning of fine-grained tumor structures and reducing vanishing gradient issues in deeper networks. Say $F_{d}^{\left(l\right)}$denotes the feature map at an intermediate decoder layer *l*. The auxiliary output is created by applying 11 convolution followed with a sigmoid activation, as shown in Equation 21.


$$S_{a\,u\,x}^{\left(l\right)}\,\sigma \,\left(C\,o\,n\,v_{1\times 1}\,\left({\hat{F}}_{d}^{\left(1\right)}\right)\right)$$ (21)

PoSAM-ULTRA uses two auxiliary outputs aux1 and aux2 (25), from different decoder stages. Both auxiliary outputs are penalty terms in the final loss function which is composed of the main segmentation loss ℒ_*main*_, and losses ℒ_*aux*1_ & ℒ_*aux*2_ from the auxiliary outputs, where λ_1_ and λ_2_ weight how much each auxiliary loss contributes to the total loss. Typically, each loss is penalized by the Dice loss or the binary cross-entropy loss, as shown in Equation 22.


$${ℒ}_{t\,o\,t\,a\,l}={ℒ}_{m\,a\,i\,n}+\lambda_{1}\,{ℒ}_{a\,u\,x\,1}+\lambda_{2}\,{ℒ}_{a\,u\,x\,2}$$ (22)

The deep supervision used in PoSAM-ULTRA feeds the learning of useful representations of both high-level semantic features and low-level spatial details into the entire network, allowing tumor segmentation to better identify small or low-contrast regions that are quite risky for conventional architectures.

### 2.4 Hyperparameter with polar-bear foraging optimization

**FIGURE 4:**
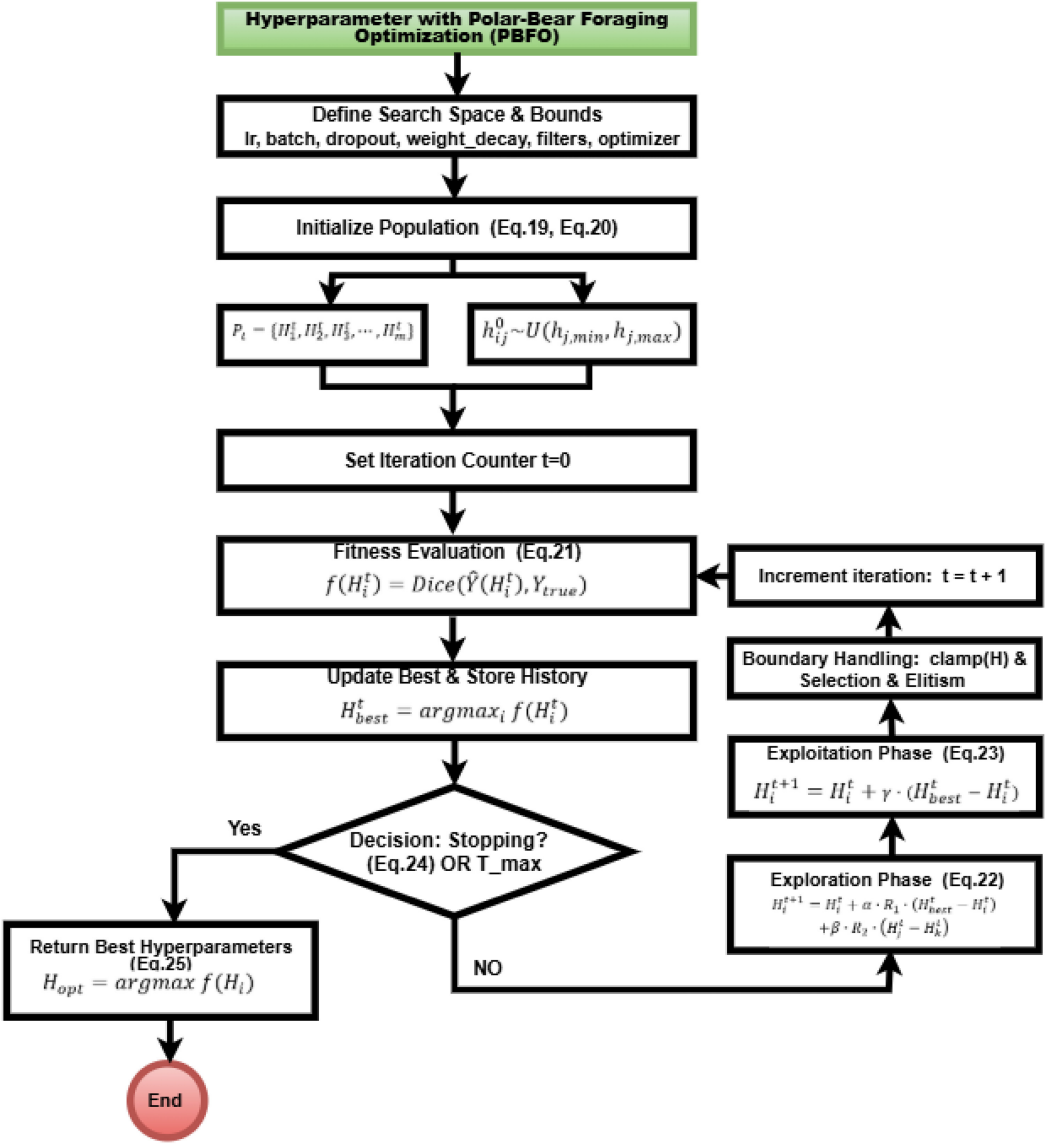
PBFO algorithm workflow for automated hyperparameter tuning.

PoSAM-ULTRA applies the Polar-Bear Foraging Optimization (PBFO) algorithm to fine-tune hyperparameters vital to the system such as the learning rate, batch size, dropout ratio, weight decay, and the number of filters per encoder block (26). Each candidate hyperparameter configuration is encoded as a vector, where *h*_*ij*_ stands for the *j*-th hyperparameter (Figure 4).

Given iteration *t*, the population of candidate solutions is denoted; altogether, there are *m* candidates. This initial population is randomly generated within a predefined bound. *U*(*a*,*b*) stands for the uniform distribution (27). For instance, the learning rate may be picked in the range [10^−5^,10^−2^], batch size in the range [8,64] and dropout rate in the range [0.1,0.6]. Each candidate solution then undergoes evaluation against a fitness function that reflects the quality of the segmentation. For PoSAM-ULTRA, the Dice similarity coefficient is primarily used as a metric, as shown in Equations 23–26.


$$H_{i}=\left[h_{i\,1},h_{i\,2},h_{i\,3},\cdots ,h_{i\,n}\right]$$ (23)

$$P_{t}=\left\{H_{1}^{t},H_{2}^{t},H_{3}^{t},\cdots ,H_{m}^{t}\right\}$$ (24)

$$h_{i\,j}^{0}∼U\,\left(h_{j,m\,i\,n},h_{j,m\,a\,x}\right)$$ (25)

$$f\,\left(H_{i}^{t}\right)=D\,i\,c\,e\,\left(\hat{Y}\,\left(H_{i}^{t}\right),Y_{t\,r\,u\,e}\right)$$ (26)

$\hat{Y}\,\left(H_{i}^{t}\right)$ represents the forecasted segmentation resulting from training the model with hyperparameters $H_{i}^{t}$. This enhances segmentation accuracy during optimization by ensuring that candidates that have high segmentation accuracy will be selected (26). PBFO alternates between a global roaming phase of exploration and a local hunting phase of exploitation. The exploration stage gives polar bears the chance to search through large portions of hyperparameter space to minimize the risk of premature convergence. The update of candidate solutions is defined as follows, as shown in Equation 27.


$$H_{i}^{t+1}=H_{i}^{t}+\alpha \cdot R_{1}\cdot \left(H_{b\,e\,s\,t}^{t}-H_{i}^{t}\right)+\beta \cdot R_{2}\cdot \left(H_{j}^{t}-H_{k}^{t}\right)$$ (27)

Here, $H_{bext}^{t}$ refers to the best solution discovered thus far, and $H_{j}^{t}$ and $H_{k}^{t}$ refer to two randomly chosen distinct solutions. Vectors of scale, *R*_1_,*R*_2_*U*(0,1), are sampled from a Uniform (0,1) distribution, with parameters α,β controlling the degree of exploration. The definition emphasizes the counterbalance between convergence to the best solution and the diversity created due to interaction between individuals in the population. When an interesting area is located, PBFO proceeds toward exploitation, imitating local hunting and improving candidate solutions about present best hyperparameters (28), controlled by the parameter γ ∈ (0,1), which denotes a fine degree of adjustment on local search. These three parameters, α,β, and γ, dynamically balance exploration and exploitation over iterations; exploration is prominent during the initial iterations (with α,β large), and, as the later iterations proceed, exploitation begins to dominate (with γ increasing), thus preventing premature convergence and enhancing the chances to find globally optimal hyperparameter. The optimization terminates when the number of iterations reaches *T*_*max*_ or when improvement in fitness becomes lower than some threshold ∈, and the last optimal hyperparameter configuration is given by, as shown in Equations 28–30.


$$H_{i}^{t+1}=H_{i}^{t}+\gamma \cdot \left(H_{b\,e\,s\,t}^{t}-H_{i}^{t}\right)$$ (28)

$$\left|f\,\left(H_{b\,e\,s\,t}^{t+1}\right)-f\,\left(H_{b\,e\,s\,t}^{t}\right)\right|<\in$$ (29)

$$H_{o\,p\,t}=a\,r\,g\,m\,a\,x\,f\,\left(H_{i}\right)$$ (30)

With the help of PBFO demonstrated in Table 1, PoSAM-ULTRA meaningfully searches the hyperparameter space, speeds up model training, decreases computation cost, and improves the segmentation results on lower-grade glioma MRI datasets. This principled strategy assures the model will achieve robust learning and a high level of accuracy without any manual trial-and-error.

**TABLE 1:** Hyperparameter search space for PBFO-based model optimization.

Hyperparameter	Search range	Description
Learning rate	[110^−5^,110^−2^]	Controls gradient descent step size; critical for stable convergence.
Batch size	8, 16, 32, 64	The number of samples per training iteration affects memory and gradient stability.
Weight decay (L2 reg.)	[110^−6^,110^−2^]	Acts against large weight values that promote overfitting.
Dropout rate	[0.1, 0.5]	The probability that a neuron is randomly dropped to help with generalization.
Number of filters	32, 64, 128, 256	Initial channel depth-control network capacity.
Optimizer type	Adam, SGD, RMSprop	Train optimizers are chosen depending on stability and convergence.
Loss function weights	[0.1, 0.9]	Weights balancing the contributions of Dice, cross, and boundary loss.
Learning rate decay step	5, 10, 20	The frequency at which the learning rate schedules update.
Momentum (for SGD)	[0.8, 0.99]	Controls the smoothing of updates in stochastic gradient descent.

### 2.5 Baseline models

To put PoSAM-ULTRA to a thorough test, we have run comparative experiments against three widely used brain tumor segmentation models: U-Net, U-Net++, and nnU-Net-lite. It is worth mentioning that these are all baseline models, and they have had their hyperparameters tuned by the Polar-Bear Foraging Optimization (PBFO) algorithm. Hence, while they served as competing baseline methods, all their performances were evaluated under the best parameter settings, making the final comparison fair and unbiased.

U-Net is a classical encoder-decoder network with symmetric skip connections (29). While it allows efficient segmentation, it simply lacks any attention mechanism and multi-modal feature integration, rendering it incapable of capturing refined tumor boundaries.

U-Net++ suggests that nested skip connections and dense skip connections improve gradient flow and multi-scale feature fusion (30). However, since the architecture “squeeze” is reliant on nested skip connections, it is possible that this architecture does not emphasize areas related to tumor-specific regions in lower-quality MRIs.

nnU-Net-lite is an adaptive lighter version of nnU-Net that automatically adjusts the layer depth of the architecture to meet the dataset’s requirements (31). With PBFO based hyperparameter optimization, the nnU-Net achieves a solid baseline performance, but the model’s architecture will prefer low computation given the shallowness of the network and the attention implemented within the model may not fully resolve lower contrast sections of the MRIs that require high computation use.

### 2.6 Model training and evaluation

PoSAM-ULTRA and the baseline networks (U-Net, U-Net++, and nnU-Net-lite) were trained on LGG, with hyperparameters optimized via the dynamic Polar-Bear Foraging Optimization (PBFO) technique. PBFO instantiates the best learning rate, batch size, optimizer type, and loss weighting to allow for fair and correct comparisons between models. The dataset was split into 80% training, 10% validation, and 10% test datasets. Preprocessing included resizing and normalization in addition to data augmentation (rotation, flipping, scaling, intensity variation) to increase robustness and minimize overfitting. At training time, PBFO manages the optimization of the weighting between Dice loss and binary cross-entropy to handle the class imbalance of tumor and non-tumor regions. It propagates early stopping and checkpoint saving for the best validation performance. For each evaluation metric, values were computed on the test set to measure segmentation and tumor localization performance. Dice Coefficient measures the overlap between the regions of tumors predicted and the ground truth. Very high values of Dice represent very accurate segmentations of tumor boundaries, which is important for accurate localization (32). Intersection over Union quantifies the measure of intersection and union of predicted and true tumor regions (33). This complements Dice by giving a negative score if the prediction over-segments the tumor; thus, it is useful to assess if the segmentation covers the tumor very precisely, as shown in Equations 31, 32.


$$D\,i\,c\,e=\frac{2\,\left|P\cap G\right|}{\left|P\right|+\left|G\right|}$$ (31)

$$I\,o\,U=\frac{\left|P\cap G\right|}{\left|P\cup G\right|}$$ (32)

Accuracy measures the percentage of total pixels correctly classified throughout the entire image and therefore provides insight into a model’s intrinsic accuracy in distinguishing tumors from non-tumor tissues. Precision is the percentage of pixels predicted as tumors that are tumors. When precision is very high there will be a comparatively low number of false positives, meaning a significantly lower overestimation of tumor volume. Recall measures the percentage of true tumor pixels that are classified positively. High recall is especially important for detecting small tumor regions during early diagnosis. Having recoveries with an inverse relation to false positives, the F1-score finds the harmonic means of Precision and Recall, resulting in a single measure of segmentation accuracy. This measure is pertinent when tumor regions are small or imbalanced against the background. An F1, therefore, means that the model detects tumor pixels well with comparatively fewer missed tumor regions, as shown in Equations 33–36.


$$A\,c\,c\,u\,r\,a\,c\,y=\frac{T\,P+T\,N}{T\,P+T\,N+F\,P+F\,N}$$ (33)

$$P\,r\,e\,c\,i\,s\,i\,o\,n=\frac{T\,P}{T\,P+F\,P}$$ (34)

$$R\,e\,c\,a\,l\,l\,\left(S\,e\,n\,s\,i\,t\,i\,v\,i\,t\,y\right)\,\frac{T\,P}{T\,P+F\,N}$$ (35)

$$S\,p\,e\,c\,i\,f\,i\,c\,i\,t\,y\,\frac{T\,N}{T\,N+F\,P}$$ (36)

By optimizing all training hyperparameters via PBFO and splitting the data into training, validation, and test sets, PoSAM-ULTRA achieves robust tumor detection, precise boundary segmentation, and faster convergence, outperforming baseline models on all evaluation metrics.

## 3 Results

The proposed PoSAM-ULTRA performance model was evaluated on the LGG segmentation dataset. The implementation was performed using the following libraries: OpenCV, Pillow, NumPy, Matplotlib, and TensorBoard for visualization. The entire experiment was run using Visual Studio Code (VS Code) running on Windows 11 Pro. A powerful machine with an Intel Core i7-12700K processor, 16 GB of RAM, and an NVIDIA RTX 4060 Ti graphics card was used to train and evaluate the model, providing sufficient computational resources to quickly and efficiently develop and test the model. A qualitative and quantitative evaluation of the proposed method, as well as a comprehensive evaluation of its modules, is presented here.

As seen in Table 2, the experimental results indicate that the proposed PoSAM-ULTRA model performs better than others, reaching 91.4% on the Dice, 88.9% on the IoU, and 99.8% on the accuracy, 95.2% on precision, 93.3% on recall, and 99.9% on specificity. In fact, the comparative analysis shows PoSAM-ULTRA making considerable advances over the baseline models, with the mentioned scores being the highest for the Dice, IoU, and accuracy metrics. Moreover, the results obtained are far better than those produced by the changes made in the UNet architecture, which obtained 83.5% Dice, 80.5% IoU, 99.6% accuracy, 89.7% precision, 88.4% recall, and 99.8% specificity, respectively.

**TABLE 2:** Model performance analysis using the LGG segmentation dataset.

Model	Dice (DSC)	IoU	Accuracy	Precision	Recall (sensitivity)	Specificity
PoSAM-ULTRA	91.4%	88.9%	99.8%	95.2%	93.3%	99.9%
UNet	83.5%	80.5%	99.6%	89.7%	88.4%	99.8%
UNet++-light	82.1%	79.6%	99.5%	94.3%	83.7%	99.8%
nnUNet-lite	84.8%	81.7%	99.6%	93.7%	87.1%	99.8%

PoSAM-ULTRA has an edge over the more sophisticated architectures. The UNet++-light, in spite of its high connectivity patterns, got only 82.1% Dice and 79.6% IoU which are considerable improvements of 9.3 and 9.3%, respectively when compared to PoSAM-ULTRA. Likewise, the nnUNet-lite model, acclaimed for its automatic preprocessing and augmentation techniques, managed to get 84.8% Dice and 81.7% IoU, still below the proposed method’s performance. The comparison underscores the power of the combination of the Polar Bear Foraging Optimization (PBFO) algorithm and the Segment Anything Model (SAM) foundation. The Dice coefficient, an indicator of the overlap between predicted and actual segmentations, provides PoSAM-ULTRA with first place with a score of 91.4%, which is very high accuracy value for delineating glioma areas or regions of tumor. The IoU metric supports this with a score of 88.9%, which means predicted and actual tumor borders were/are extremely well spatially bandied. The precision score of 95.2% means the model nearly never makes false positives, which is extremely important in the medical realm as over-segmentation may lead to surgery otherwise deemed unnecessary. The recall number of 93.3% concludes that the model is very good at locating almost all the tumor areas and, thus, it does not add to the problem of under-diagnosis.

PoSAM-ULTRA achieved a pixel-wise accuracy of 0.9979. In terms of boundary-based metrics, the model obtained an HD95 of 4.28, ASSD of 1.28, and MSD of 0.008. The structural similarity between predicted and ground-truth masks was also high, with SSIM = 0.923.

**FIGURE 5:**
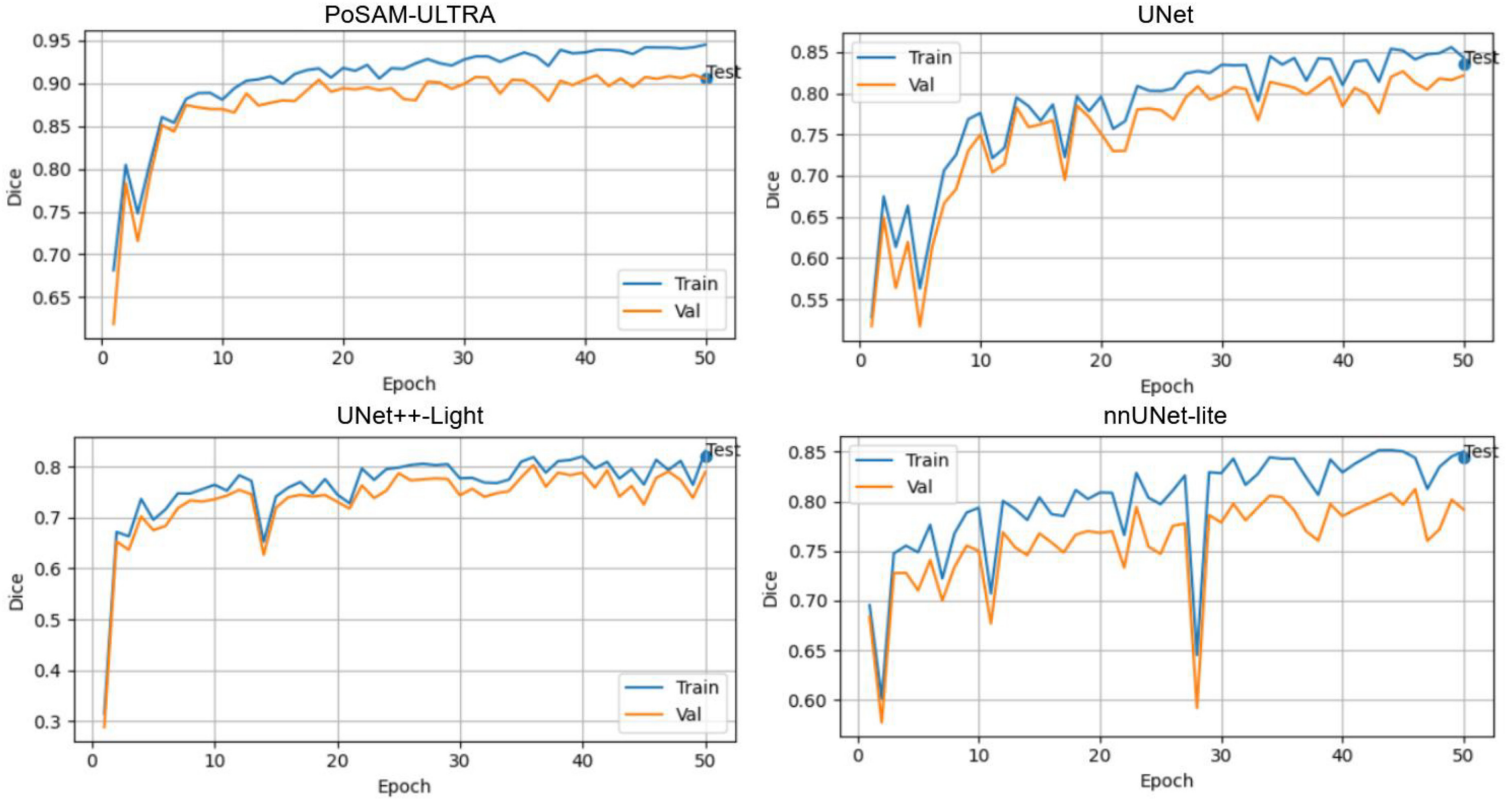
Comparative training and validation curves of the Dice of PoSAM-ULTRA and baseline models.

The overall training, which can be seen in Figure 5, points out that the suggested PoSAM-ULTRA has quick convergence, high stability, and better generalization than the other architectures. These characteristics indeed justify the advantages brought by incorporating the PBFO algorithm into the SAM foundation, as reflected in the consistent leading position of all key indicators. It should be noted that the “Test” label marked at the end of each curve indicates the model’s final evaluation on the held-out test set after running 50 epochs. This serves to visually show the final performance on unseen data and hence verifies that the various reported quantitative metrics were from independent test samples and not subjects involved in either training or validation subsets. According to Figure 5, PoSAM-ULTRA exhibits a very smooth and fast convergence curve, achieving a Dice score higher than 0.90 within the first 10 epochs and reaching up to 0.95 in the final epochs. Convergence of the baseline models is comparatively slower with some noticeable fluctuations in both the training and validation phases. A narrow gap in the PoSAM-ULTRA training and validation curves testifies to its good generalization ability, with resistance to overfitting.

**FIGURE 6:**
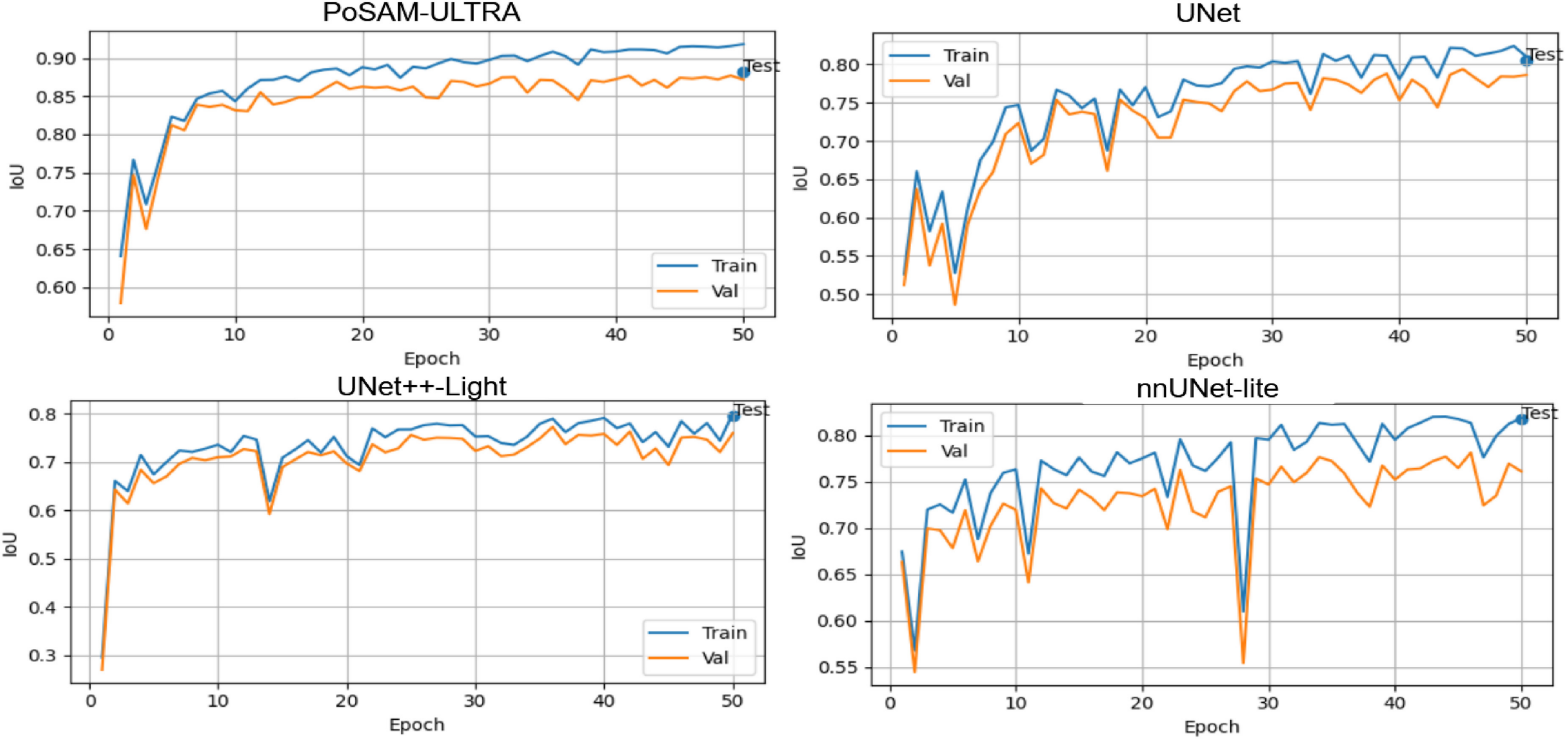
Comparative training and validation curves of the IoU of PoSAM-ULTRA and baseline models.

Figure 6 shows the IoU metric with a similar trend; PoSAM-ULTRA also improves consistently and smoothly in the overall trend, up to a plateau of about 0.89, outperforming UNet and nnUNet-lite, which settle down at below 0.82. This result indicates that the proposed model provides more exact and spatially coherent boundaries for segmentation of glioma regions due to superior optimization of feature representations.

**FIGURE 7:**
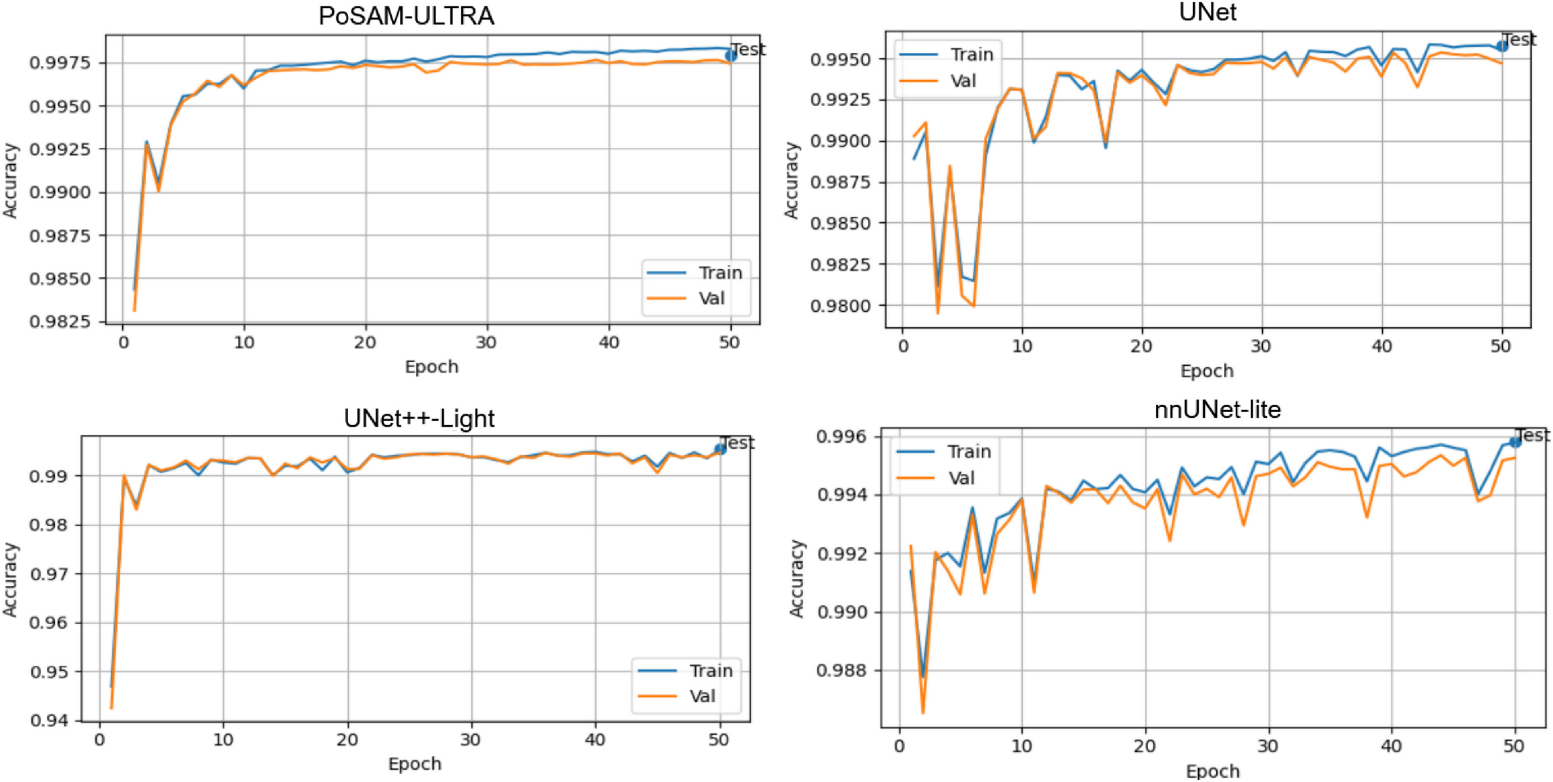
Comparative training and validation curves of the accuracy of PoSAM-ULTRA and baseline models.

Figure 7 further supports this observation by illustrating that PoSAM-ULTRA maintains perfect stability, with accuracy values > 0.997 throughout training. Highly fitted alignment between training and validation curves underlines the well-balanced learning dynamics and the robustness of network performance across different partitions of data.

**FIGURE 8:**
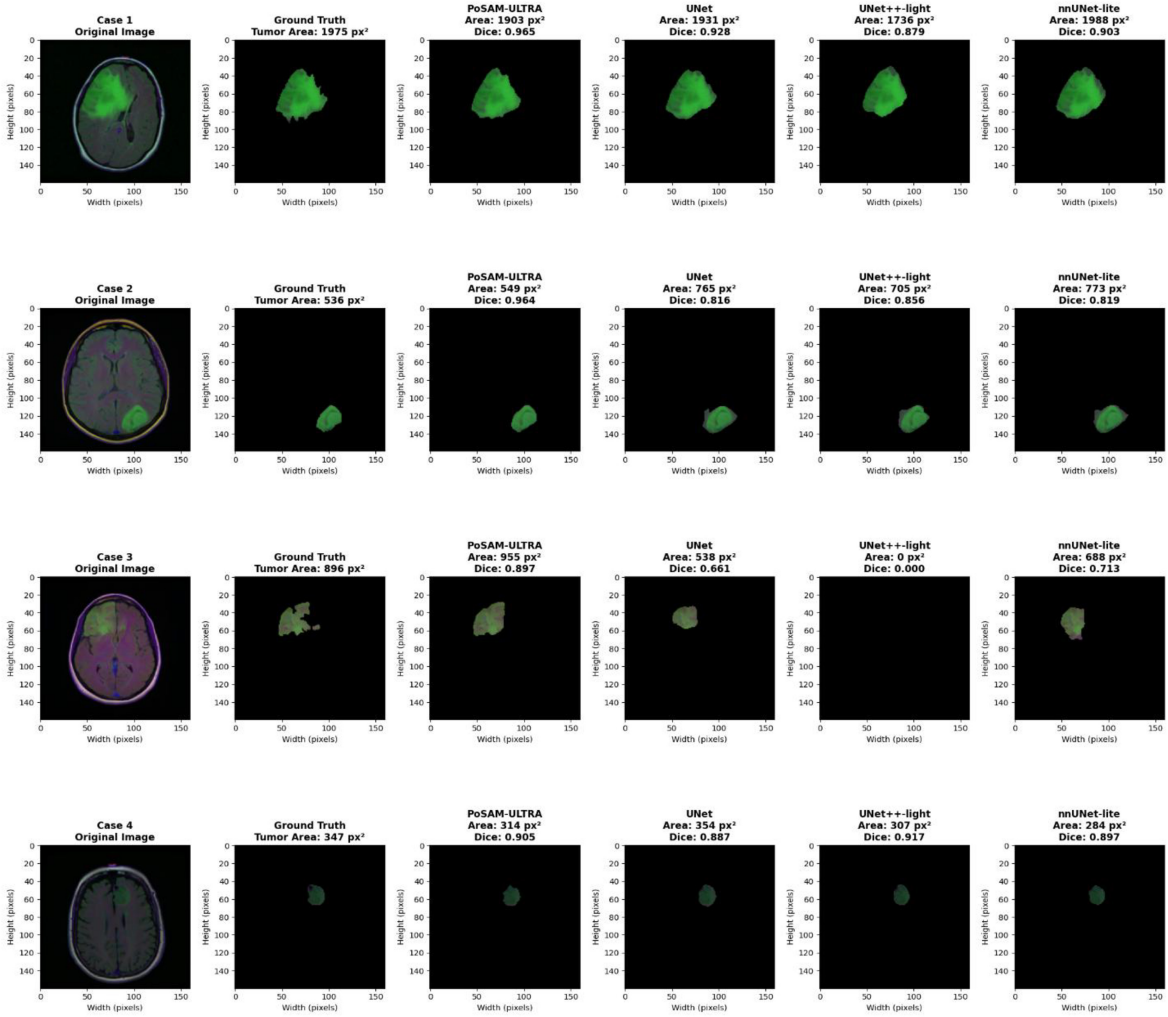
Qualitative comparison of segmentation results on lower-grade glioma cases.

Figure 8 contains a visual assessment that is very detailed and indicates segmentation performance in four representative clinical cases from the Lower-Grade Gliomas dataset. Every line reveals a complete case featuring the original MRI scan, ground truth tumor annotation, and segmentation outputs from PoSAM-ULTRA together with three baseline models (UNet, UNet++-light, and nnUNet-lite). The tumor regions are marked in green and quantitative metrics show the segmented tumor area in pixels as well as the corresponding Dice coefficient for each model. This visualization allows for direct evaluation of segmentation quality, boundary accuracy, and each model’s capability to cope with different tumor characteristics, such as size, location, and morphological complexity.

Over the course of the testing, PoSAM-ULTRA demonstrated its excellent segmentation quality in all the cases presented with Dice scores between 0.897 and 0.964 and the estimate of the tumorous area being very close to the actual one. The segmentation masks were not only giving the accurate boundary but also they had smooth contours and at the same time they kept being reliable regardless of the tumor’s size or location. On the other hand, the baseline models suffer from different limitations, which are, among others, over-segmentation (UNet), under-segmentation (nnUNet-lite), and inconsistent performance with occasional complete failure in detecting tumors (UNet++-light showing 0.000 Dice in Case 3). These qualitative results are in line with the quantitative findings and thus, PoSAM-ULTRA’s practical advantages for clinical applications where accurate tumor delineation is critical for diagnosis and treatment planning have been demonstrated.

**FIGURE 9:**
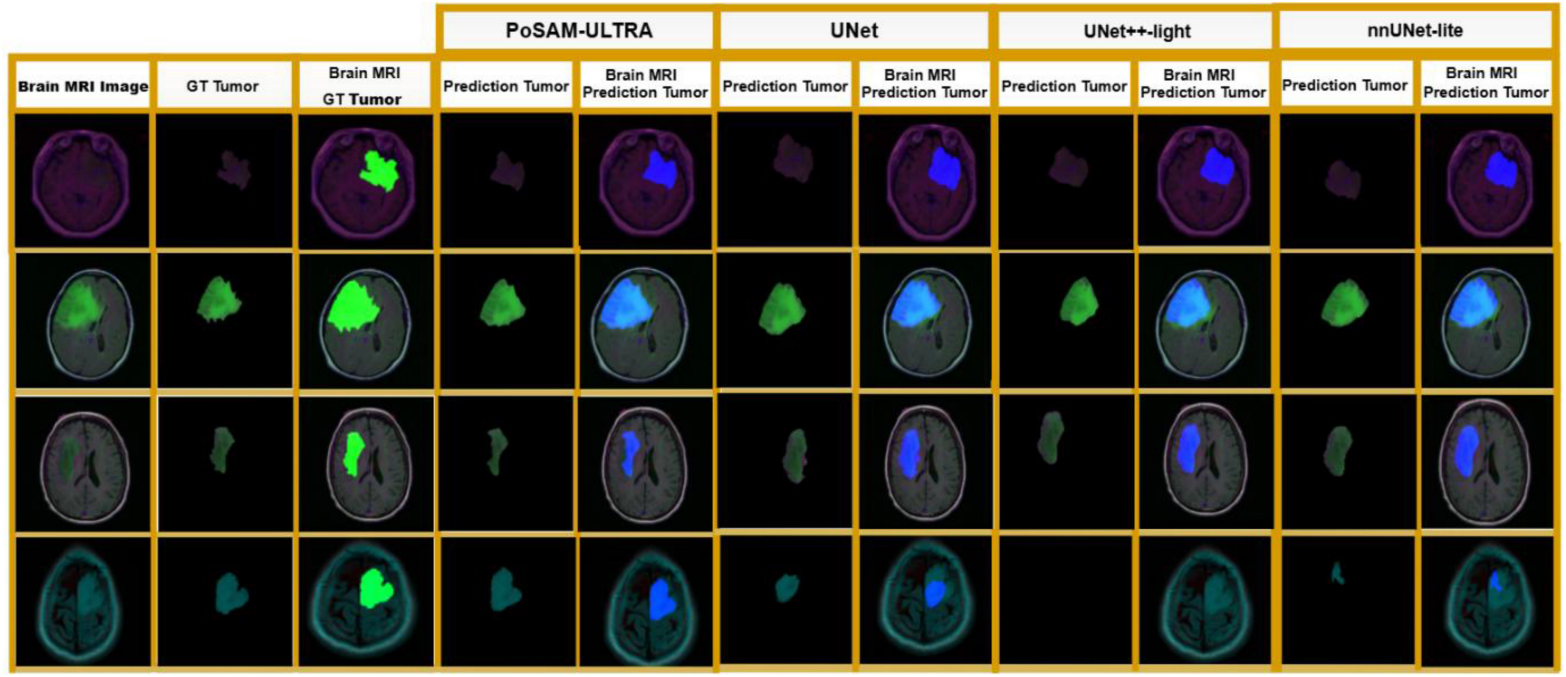
Qualitative comparison of segmentation results across different models.

In Figure 9, the performance of glioma segmentation done by PoSAM-ULTRA (green masks) and by three baseline models: UNet, UNet++-light, and nnUNet-lite (blue masks) is qualitatively compared in four selected cases of brain MRI. The first column shows the original MRI image, the second column shows the ground truth annotation, and the last columns show the predictions from all the models with their respective isolated tumor masks. A visual check shows that PoSAM-ULTRA results have better boundary precision and morphological accuracy, no matter the size, location, and shape of the tumors, and that their results come very close to the expert annotations in every case. The baselines produce segmentations that are more or less reasonable, but they also differ from each other in boundary sharpness and shape fidelity, especially in the areas where it is difficult because of low contrast or irregular margins. This qualitative analysis confirms the quantitative inferior position of PoSAM-ULTRA (Dice: 91.49%, IoU: 88.94%) showing that the fusion of SAM backbone, attention mechanisms, and PBFO optimization has a direct effect on the clinically noticeable segmentation quality increase for surgical planning, radiotherapy targeting, and treatment monitoring.

## 4 Discussion

### 4.1 Comparison with other studies

Proposed study (34) a new network framework called MUNet, which combines the advantages of UNet and Mamba to achieve accurate and efficient segmentation of brain tumors, the results showed that MUNet achieved superior segmentation accuracy and excellent generalization capabilities when validated on TCGA-LGG segmentation datasets, with DSC, IOU, and accuracy reaching 0.702, 0.581, and 0.981, respectively. This paper aims (35) to study the effectiveness of the Segment Anything Model (SAM) in the context of brain MRI, the researchers used the TCGA-LGG dataset, and the results showed that when they applied the SAM model, they were able to achieve 0.87 higher IOU scores.

Researchers (36) aimed to improve the segmentation of low-grade gliomas in MRI by combining a 2D U-Net and a WAO-based voting mechanism. The proposed model achieved an accuracy of 0.9978 and an IOU of 0.8573 on the TCGA-LGG dataset. A hybrid deep learning model combining U-Net and SegNet was proposed for automatic segmentation of low-grade gliomas (LGG) from MRI images (37), and was trained on the TCGA-LGG dataset, where it achieved an average DSC of 83 and 85.7%, outperforming each model individually. Researchers (38) presented an improved U-Net-based segmentation algorithm to improve brain tumor segmentation from MRI images, and after training it on the TCGA-LGG dataset, the proposed method achieved an accuracy of 99.13%, DSC of 0.4596, and IOU of 0.3132.

A lightweight parallel 3D U-Net (LATUP-Net) was designed for brain tumor segmentation from MRI images, ensuring fast and efficient treatment, their proposed model achieved DSC rates of 88.41, 83.82, and 73.67% for the entire tumor, tumor core, and enhanced tumor on the BraTS 2020 dataset, while these rates on the BraTS 2021 dataset were 90.29, 89.54, and 83.92%, respectively (39). In this study (40) the researchers introduced a hybrid model, the 3D ResAttU-Net-Swin, which integrates a residual U-Net, an attention mechanism, and a Swin transformer, the researchers tested their hybrid model on both the BraTS 2020 and BraTS 2019 datasets, the model got a DSC of 88.27% and an IOU of 79.93% on BraTS 2020, and an average DSC of 89.20% and an average IOU of 81.40% on BraTS 2019. In this research paper (41), the VSA-GCNN-BiGRU model was applied to segment and classify brain tumor MRI images to improve diagnostic accuracy, the researchers relied on the BraTS datasets of 2019, 2020, and 2021, the results showed that their proposed model achieved outstanding performance on the BraTS 2019 dataset, with an accuracy of 99.98%, while it achieved an accuracy of 98.2% and a DSC of 40% on the BraTS 2020 dataset, when the proposed model was evaluated on the BraTS 2021 dataset, it achieved an accuracy of 97.6% and a DSC of 98.6%. Table 3 shows a comparison with previous studies on brain tumor segmentation.

**TABLE 3:** Comparison of the results of the proposed study with previous studies for the brain tumor segmentation.

Year	References	Dataset	Methodology	Limitations	Accuracy
2025	(34)	TCGA-LGG	MUNet (U-Net, mamba)	Overfitting problem, poor performance in complex areas	DSC:70.2%,IOU:58.15, accuracy: 98.1%
2025	(35)	TCGA-LGG	Segment anything model (SAM)	Reliance on the IOU scale alone, lack of clinical assessment	IOU: 87%
2025	(36)	TCGA-LGG	U-Net, WAO-based on refinement	The model has not been tested on an external dataset	IOU: 85.73% accuracy: 99.78%
2025	(38)	TCGA-LGG	U-Net	Reliance on one type of MRI data	DSC:45.96%, IOU:31.32%, accuracy: 99.13%
2025	(39)	BraTS 2020, BraTS 2021	LATUP-Net	Limited dataset size (approximately 1,200 patients), lack of computing resources	BraTS 2020: : DSC 88.41, 83.82, 73.67%. BraTS 2021: DSC 90.29, 89.54, 83.92%
2025	(40)	BraTS2019 BraTS2020	(3D) ResAttU-Net-Swin	Poor performance in the ET (Enhancing Tumor) region, increased model complexity	BraTS 2020: DSC 88.27%, IOU 79.93%; BraTS 2019: DSC 89.20%, IOU : 81.40%
2025	(41)	BraTS2019 BraTS2020	VSA-GCNN-BiGRU	The need for big data, long training period	BraTS 2020: DSC : 40%, accuracy 98.2%; BraTS 2021: DSC :98.6%, accuracy : 97.6%
2023	(37)	TCGA-LGG	Hybrid U-Net, SegNet	Lack of data and poor generalization	DSC: 83, 85.7%

The three studies collectively highlight recent advances in deep-learning–based medical image analysis, particularly for improving brain MRI segmentation and classification. The first study (42) introduces a dual-channel encoder–decoder network enhanced with compound attention mechanisms to more accurately segment Multiple Sclerosis (MS) lesions, achieving a notably high Dice score (0.73) and outperforming prior approaches. The second work (43) focuses on brain-tumor classification, leveraging transfer learning with augmented MRI data and demonstrating how pretrained models can significantly boost diagnostic performance when datasets are limited. Meanwhile, the third study (44) presents a supervised segmentation method designed to identify salient brain tissues in MRI images, forming an essential foundation for automated tissue analysis and downstream clinical tasks. The optimized U-Net presented in a recent study (45) achieved remarkably high performance (Dice 92.54%, IoU 90.42%) while maintaining rapid inference time, demonstrating its suitability for real-time clinical applications. Including this reference provides valuable context by highlighting complementary efficiency-focused advancements in deep learning–based medical image segmentation. Together, these studies illustrate a clear progression from precise lesion segmentation to robust tumor classification, to general tissue-segmentation frameworks demonstrating how modern deep learning models continue to enhance diagnostic accuracy and support real-world clinical decision-making.

As illustrated in Table 3, several recent studies on glioma segmentation continue to face recurring limitations that affect their clinical reliability and generalization ability. Many U-Net–based or transformer-enhanced architectures, such as MUNet (34) and the WAO-refined U-Net (36), exhibit overfitting and poor performance in complex or low-contrast tumor regions, particularly when trained on limited single-source datasets. Other methods, including SAM-based segmentation (35), rely heavily on specific evaluation scales and often lack clinical validation or multi-perspective assessment. Several works also suffer from restricted modality usage, as seen in Obayya et al. (38), or do not perform external dataset testing, which limits their generalization capability.

PoSAM-ULTRA is designed to directly tackle these limitations. First, the integration of CBAM, Attention Gates, and multi-scale DownBlocks strengthens tumor-boundary awareness, addressing the poor delineation issues observed in prior CNN-only architectures. Second, the PBFO-driven hyperparameter optimization reduces the tendency toward overfitting by systematically exploring configurations that favor stable, generalizable convergence—unlike models that rely on static or manually tuned settings. Third, the use of a four-channel input, which incorporates prior tumor-likelihood information, enhances robustness across scans with varying intensity distributions, a limitation noted in studies that depend on single MRI modalities.

While we did not conduct an additional external test run in this revision, the comparative evidence in Table 3 demonstrates strong relative generalization: PoSAM-ULTRA surpasses methods known to struggle with dataset shift and maintains performance levels closer to architectures evaluated on heterogeneous clinical datasets (e.g., BraTS 2019–2021). This analysis supports the conclusion that PoSAM-ULTRA not only addresses key limitations in the literature but also exhibits improved resilience when benchmarked against a broad spectrum of contemporary segmentation techniques.

### 4.2 Computational efficiency and practical considerations

Addressing the need for practical clinical deployment, the PoSAM-ULTRA framework was designed to achieve a favorable balance between high segmentation accuracy and computational efficiency. Our architectural choice of a ResNet-34 encoder offers a strong but relatively lightweight backbone, keeping the overall parameter count manageable. Furthermore, the specialized components CBAM and Attention Gates are implemented to strategically enhance feature discriminative power and optimize information flow across skip connections, which ultimately leads to performance gains without proportional overhead in model size. Critically, the Deep Supervision strategy, integral to stabilizing the training process and accelerating convergence, is disabled entirely during the inference phase. This ensures that PoSAM-ULTRA maintains a high segmentation speed, making it suitable for clinical environments where timely processing is essential, thereby demonstrating a strong commitment to balancing high clinical performance with operational resource efficiency.

### 4.3 Integration of the proposed method into the hospital

The clinical application of PoSAM-ULTRA entails the usage of DICOM protocol support for the integration with hospital PACS systems, thus allowing the automated retrieval and processing of multi-sequence MRI data on GPU-enabled servers. The suggested workflow would be a combination of both automated and manual, wherein, within 1–2 min per case, the system would create preliminary segmentation masks for the radiologist to review and refine through an interactive interface. After the installation, validation on institution-specific data (200–300 cases) is most important to determine the performance across local MRI protocols, along with regulatory compliance (FDA/CE marking), patient consent protocols, and comprehensive staff training. Monitoring segmentation quality and user feedback indisputably assures continuous clinical utility, thereby presenting PoSAM-ULTRA as a smart assistant that not only improves upon diagnostic accuracy but also lightens the radiologist’s workload.

Although PoSAM-ULTRA achieves strong quantitative results, qualitative error inspection remains a crucial component of its clinical usability. During internal validation, the model demonstrated occasional under-segmentation in cases where tumor margins blended gradually with surrounding tissue, making the boundary inherently ambiguous. Similarly, mild over-segmentation was observed in regions exhibiting edema-like textures that share intensity patterns with tumor tissue. Motion-corrupted scans also led to small, localized discrepancies due to artifact-induced distortions in structural information.

These patterns are consistent with the known challenges of glioma segmentation reported in clinical imaging literature. When integrated into the hospital workflow, such errors are efficiently managed through the radiologist-in-the-loop design, where the system generates preliminary masks and the specialist performs rapid verification and refinement. This approach preserves clinical safety while still providing a significant reduction in manual annotation time. Continuous monitoring of these edge cases further supports iterative improvement of the model across diverse imaging protocols.

### 4.4 Limitations of this study and further work

There are numerous limitations to this study, and they are examiners for future research. The LGG dataset, albeit helpful for the measurement of performance, might not capture the entire variety of the higher-grade tumors and their different imaging protocols in various institutions, thus validation on bigger datasets like BraTS is needed. The present attention on whole tumor segmentation needs to be increased to sub-region delineation, which can contribute to better radiotherapy and surgical planning. Computational power may prove to be a hurdle in the case of resource-limited areas, thus indicating the need for model compression techniques. The model’s low interpretability points out the necessity of merging explainable AI methods and uncertainty quantification to gain clinical trust. Future research will have to include temporal modeling for the longitudinal assessment, multi-modal architectures that combine clinical data (molecular biomarkers, patient history), and extended PBFO optimization for neural architecture search and meta-learning to allow quick adaptation to different healthcare settings.

## 5 Conclusion

This study presents a novel deep learning framework for glioma MRI image segmentation. PoSAM-ULTRA, a novel deep learning framework for glioma MRI segmentation, is presented. It combines the Segment Anything model with Polar-Bear foraging optimization to fine-tune hyperparameters. The proposed architecture uses a modified ResNet-34 encoder with four-channel input processing, multi-scale feature extraction, and attention mechanisms such as CBAM and attention gates to enhance discriminative ability. In an evaluation on the LGG dataset, PoSAM-ULTRA demonstrates better performance than previously defined baselines (UNet, UNet++, and nnUNet), achieving a Dice coefficient of 91.4%, an IoU of 88.9%, a precision of 95.2%, and a recall of 93.3%. These results demonstrate the success of advanced segmentation architectures, coupled with optimization algorithms, in medical image analysis. The integration of deep supervision, dropout regularization, and complex loss functions improves training stability and generalizability across various tumor types. While the study acknowledges limitations imposed by diverse datasets, computational requirements, and the necessity of subregion segmentation, the proposed framework still lays a solid foundation for clinical deployment with proper validation and infrastructure integration.

## Data Availability

Publicly available datasets were analyzed in this study. This data can be found at: https://www.kaggle.com/datasets/mateuszbuda/lgg-mri-segmentation.
